# The Influence of a Celebrity Chef on Customer Repurchase Behavior: Empirical Study of Taiwan’s F&B Industry During COVID-19 Pandemic

**DOI:** 10.1177/21582440231174177

**Published:** 2023-05-30

**Authors:** Hwai-Shuh Shieh, Shu-Chen Lin

**Affiliations:** 1 Shih Chien University, Taipei, Taiwan; 2 Kainan University, Taoyuan, Taiwan

**Keywords:** corporate brand, celebrity chef, repurchase behavior, food and beverage industry, COVID-19

## Abstract

The spread of COVID-19 pandemic has inflicted severe blows on the global hospitality industry. In Taiwan, revenue from the food and beverage (F&B) department has decreased by more than 90%. This study aims to understand whether celebrity chefs can effectively help and enhance their corporates’ business performance under COVID-19′s severe impacts via leveraging their personal brand value, explores the influence of a celebrity chef on customer repurchase behavior during the epidemic and examines whether such a chef has a mediation effect on the relationship between corporate brand and customer satisfaction. The primary data were collected from the respondents through online questionnaire in Taiwan to get 245 respondents as a sample size of the research from Nov. 10 to Nov. 25 in 2021, and through validity and reliability analysis that processed by statistical software using factor analysis and structural equation modeling to see if celebrity chefs’ personality branding could influence customer repurchase behavior, and also examine the relationship between corporate brand and celebrity chef. The findings show that corporate brand enhances both a celebrity chef’s personal brand and customer satisfaction, and that a celebrity chef has a positive effect on both customer satisfaction and loyalty, which can partially mediate the effect of corporate brand; furthermore, a celebrity chef has a positive effect on customer repurchase behavior. In Taiwan relative studies into aspects of a celebrity chef’s effect on consumer behavior are limited, and so this research offers new insights into the celebrity chef phenomenon there as well as elsewhere.

## Introduction

According to World Health Organization’s statistics, the COVID-19 epidemic has affected more than 188 countries and regions around the globe. Due to severe travel restrictions, home isolation, social distancing, and global events being canceled or postponed, the spread of COVID-19 pandemic has inflicted severe blows on the global tourism and hospitality industry. An open letter from Gloria Guevara, President and CEO of the World Travel and Tourism Council, expresses that the travel and tourism sector is “already facing collapse” and is “in a fight for survival” due to the COVID-19 global health crisis (Guevara, 2020). Furthermore, compared to previous events such as natural disasters or epidemics, the impact of COVID-19 has been unprecedented (Pillai et al., 2021). Rivera (2020) stated that COVID-19 has clearly invaded the core of the hospitality industry that was among the first industries affected by the pandemic, and it seems that it will be the last to recover from it (Cajner et al., 2020).

The hospitality industry is susceptible to threats caused by unexpected catastrophes such as epidemics, natural disasters, and terrorist attacks (Chan & Lam, 2013; M. H. Chen, 2011; Huang & Rust, 2018; Jayawardena et al., 2008; Lo et al., 2006; Min et al., 2009; Paraskevas, 2013; Racherla & Hu, 2009), and Taiwan is not an exception. Because it is one of the two revenue resource for international tourist hotels in Taiwan, revenue from food and beverage (F&B) department has decreased by more than 90%. According to statistics of the Taiwan Tourism Bureau (2021), the F&B and hotel industries were the first two to be negatively impacted by the pandemic (M. H. Chen et al., 2007; Jiang & Wen, 2020).

In modern human history, people have faced many epidemic outbreaks such as Ebola, SARS, MERS etc., and most outbreaks have had a greater impact on consumer behavior (Laato et al., 2020). Although the scale and scope of pandemics’ effects vary from country to country, COVID-19 is a global health crisis that is already having a larger influence on the world economy and consumer’s behavior regarding the purchase and consumption of food. Moreover, supply chains throughout the whole world have forever been altered. Consumers are now changing their preferences for products, their lifestyle, and business environment. During the COVID-19 pandemic, people are being forced to change their consumption patterns because of home-based work, social distancing, and lockdowns, and the use of digital technology has also increased significantly, for example, videoconferencing, online chats, and use of social media (Cambefort, 2020). With COVID-19 and its variants still widely continuing to spread, some studies have been able to illustrate a significant impact on consumer behavior (Ben Hassen et al., 2020; Campbell et al., 2021; Loxton et al., 2020). Campbell et al. (2021) stated that consumer behavior during the pandemic presents a change from offline to online.

At this time most countries have chosen a different strategy in the F&B industry to prevent the spread of COVID-19, for example, keeping restaurants and bars open under service restrictions, or implementing heavier restrictions (Granberg et al., 2021). Under the circumstance, this study aims to understand whether celebrity chefs can still effectively help and enhance their corporates’ business performance under COVID-19′s severe influence by leveraging their personal brand value. Food television programs and celebrity chefs have particularly played a central role in mainstream media in recent years (Lewis & Huber, 2015). Food television programs and shows operate as platforms for the production of celebrity food brands and food businesses, which are also crucial to the success of mainstream food brands and businesses (Phillipov, 2017). Celebrity chefs’ power is cultivated by both printed and digital media (Henderson, 2011), and their professional profiles drive the consumption and the audience’s interest (Matta, 2019). Celebrity chefs share some defining characteristics and activities in spite of individual differences, and the trend can be seen as an example of globalization (Henderson, 2011).

The influence of celebrity chefs is certainly apparent in the more economically advanced regions of Asia, as demonstrated by related developments in Taiwan. McCormick (2016) indicated that celebrity chef as endorser can increase purchase intent and create favorable attitudes toward the corporate brand. Some studies showed that celebrity chef’s brands can positively influence consumer WTP, and intentions to purchase and to repurchase (Y. S. Chen et al., 2017; Ilicic & Webster, 2016; Jin et al., 2023). Before the COVID-19 epidemic, Taiwan also has some celebrity chefs, and by giving differential services and creating their own unique specialty, they have built up their personal brands to promote corporates’ business performance and enhance their own personal brand value. Reviewing the past literature, few studies focused on the relationship between the celebrity chef and intentions to repurchase during the epidemic. Therefore, the study aims to explore the influence of a celebrity chef on customer repurchase behavior during the COVID-19 epidemic and to fill up this research gap and also examines whether a celebrity chef has a mediation effect on the relationship between corporate brand and customer satisfaction, especially during the COVID-19 epidemic.

## Theoretical Background

### Corporate Brand

Corporate brand potentially has a rich heritage, assets and capabilities, people, values and priorities, a local or global frame of reference, citizenship programs, and a performance record (Aaker, 2004), which can be viewed as a type of corporate culture (Balmer, 2013; Baumgarth & Schmidt, 2010; Evans et al., 2012; Schmidt et al., 2017) and shared values, beliefs, and behaviors (Chatman & O’Reilly, 2016; Schwartz & Davis, 1981).

As a mark of assurance, a corporate brand is underpinned by the organization’s core values (Balmer, 2013; Hatch & Schultz, 2001, 2003), grows out of and represents an organization’s corporate identity (Balmer & Podnar, 2021) and delivers a corporate brand promise (Balmer, 2012a, 2012b). Corporate culture values can shape corporate brand by creating a stronger sense of brand identity and loyalty for the company. Corporate brands should meet the demands and needs of all stakeholders, including customers, and must take their perceptions of said brands into consideration (Balmer et al., 2020). Employees are a key corporate brand management constituency (Balmer, 1995), deliver and enhance brand value via their actions and behaviors (Burmann et al., 2009; Harris & de Chernatony, 2001) and ensure that customers and other stakeholders experience greater uniformity (Löhndorf & Diamantopoulos, 2014). Overall, corporate brands can positively and significantly influence consumer behaviors and more specifically by helping them make better food decisions.

### Celebrity Chefs

The term celebrity chef is associated with chefs and cooks who have become well-known through television and cookbooks (Lengyel, 2021). With the rapid development of mass communication, celebrities refer to people who have a certain amount of fan of their own on various network platforms and those who have been continuously outputting professional knowledge for a long time (Wu & Liu, 2021). Over the past two decades, there has been a surge in popular interest toward celebrity chefs (Giousmpasoglou et al., 2020), accompanying an expansion and popularity of TV lifestyle programing (e.g., Alkon & Grosglik, 2021; Barnes, 2017; Ferguson & Zukin, 1998; Henderson, 2011; Lewis & Huber, 2015; Martin, 2022; Maughan et al., 2017; Newman & Witsell, 2021). A celebrity chef is a modern cultural symbol that reflects contemporary attitudes toward cooking, consumption, and culinary taste and within this arena has become a vital part of contemporary society in transmitting concepts of taste through various media. Such chefs have some influence in transmitting messages of lifestyle and food choices through the Internet, television shows, and cookbooks. Abbots (2015) indicated that celebrity chefs foster an intimate relationship with consumers, called para-social relations by Barnes (2017), and these relations are social and economic, in which the fostering of intimacies can influence commercial relations.

Celebrity chefs have become trusted sources of information for the popular attention bestowed upon them (Furedi, 2010) as well as ordinary experts providing advice on food (Powell & Prasad, 2010). Calvo-Porral et al. (2021) stated that consumers are the most influenced in their food consumption behavior, by not only their food purchase intention, but also their willingness to pay a premium price for food, by the congruence between celebrity endorsements and the products being recommended, and by the celebrities’ credibility. Powell and Prasad (2010) noted that the influence of celebrity chefs on consumer trends in terms of food choices has grown in the last two decades. As a result of their “celebritization,” a process that commodities oneself and embodies personal characteristics (Bridgen, 2011), celebrity chefs have become commodities themselves and transformed their names into brands (Henderson, 2011). Celebrity chefs build an online personal brand by themselves, form meaningful and mutually beneficial work-related relationships, and then become part of their employer’s product and are responsible for organizational success (Bridgen, 2011). By becoming media personalities and brands (Giousmpasoglou et al., 2020), celebrity chefs enhance their restaurant’s competitive advantage and ensure commercial success in a highly competitive restaurant trade (Giousmpasoglou et al., 2020). Accordingly, it is proposed:

*H1: Corporate brand has a positive effect on celebrity chef.*

### Branding in the F&B Industry as the Context of Our Analysis

Some studies have illustrated that a product brand plays a vital role and has an impact on the perception of consumers’ preferences in the F&B industry (e.g., Ayanwale et al., 2005; Chryssochoidis et al., 2007; Espejel et al., 2007; Krystallis et al., 2007; Vraneševic’ & Stančec, 2003; Wang, 2013). Bartsch et al. (2016) indicated that the perceived brand quality is the ability to build a strong association between the brand and its values in the consumer’s mind, and they are also seen as key drivers of brand equity (Davcik, 2013). Previous F&B studies mostly explored online engagement (Pletikosa Cvijikj & Michahelles, 2013) and food experiences (Chhabra et al., 2013). In fact, F&B experiences are increasingly becoming the object of sharing on social networks (Gensler et al., 2015).

It is widely known that branding is important and has effects on firm performance. The study of Hong and Diep (2016) provided evidence for the positive relationship between branding management and business performance in Vietnamese F&B small and medium-sized enterprises. H. Kim et al. (2003) also indicated the positive effects of brand loyalty, brand awareness, and brand image on financial performance. Food industry firms usually use a branding technique to build their corporate image to attract consumers and promote sales of their products (Keller et al., 2012). Wiyana et al. (2021) suggested integrating branding strategy in developing culinary businesses to help attract customers with creative branding models. Abdillah and Kahalani (2020) stated that co-branding has a positive and significant influence on purchasing decisions and can improve sales performance. During the last decade, brands have discovered social media as a vehicle for marketing communications, and major F&B companies are using various popular social media to reach and communicate with their customers who share some common interests toward their brand (Lakshmi & Sengottuvelu, 2020).

### Customer Satisfaction in the F&B Industry

The F&B industry is a highly competitive market, and customer satisfaction is very crucial to help a business succeed. UNWTO has defined satisfaction as “a psychological concept involving the feeling of well-being and pleasure that results from obtaining what one hope from an appealing product and/or service” (Riämmiängton & Yüksel, 1998, p. 39). Westbrook and Reilly (1983) proposed that customer satisfaction is an emotional response to the experiences provided by, or associated with, particular products or services purchased. In other words, customer satisfaction is the difference between post-purchase experience and prior attitude with respect to the brand choice in question (Malhotra et al., 2016). According to Parasuraman et al. (1985), customer satisfaction is the result of a comparison between expectations and the performance achieved; that is, satisfaction is measured by comparing customers’ expectation of a product or service to their actual experience. Ho et al. (2021) illustrated that customer satisfaction mediates the relationships between predictors and repurchase intention. Theoretically, customer satisfaction and customer loyalty are considered to have a positive relationship toward each other, as presented by other scholars.

As mentioned above, a great level of customer satisfaction leads to a massive improvement in marketing productivity, including customer retention and profitability. With the support of the Internet and online social network development, corporates are finding it easier to establish and strengthen stable relationships with existing and potential customers directly and individually and thus enhance business performance. A F&B service provider can use various social media to strengthen its brand value, enhance customer satisfaction, and improve its business performance. However, every customer nowadays has the power to control the reputation of a restaurant with the help of social media. Especially with the F&B industry, reviews, recommendations, and even rumors spreading on the Internet are information that most customers are very liable to access. The sharing of information on these online platforms can spread rather fast, and so F&B businesses have to make more effort on valuing every single customer’s opinion and experience to maintain their brand image and level of customer satisfaction. Enhancing customer satisfaction by keeping customers’ engagement via social networking services is an important way in the F&B industry. With the popularity of social media, more and more consumers are willing to share their experiences by using platforms to discover F&B trends, especially younger people, who consider platform as a powerful medium in their decision-making.

Many factors influence customer satisfaction including restaurants managed by celebrity chefs (Bisui et al., 2021), and satisfaction of a culinary experience is complex as it involves various tangible and intangible elements (Chatterjee & Suklabaidya, 2021). Some studies have shown that consumers’ satisfaction in fine-dining restaurants relies more on the chefs’ food arrangement (Cifci et al., 2023; Correia et al., 2008; Namkung & Jang, 2007), whereas a celebrity endorsement has more significant effects on customer satisfaction (Legendre & Baker, 2021). Based on this it is affirmed:

*H2: Corporate brand has a positive effect on customer satisfaction.*
*H3: Celebrity chef has a positive effect on customer satisfaction and has a mediation effect in the relationship between corporate brand and customer satisfaction.*

### Repurchase Behavior and Customer Loyalty

Repurchase intention is an individual’s judgment about purchasing again from the same company (Hellier et al., 2003); that is, it is a repeat intention to buy the product or service (C. W. D. Chen & Cheng, 2009; Khalifa & Liu, 2007; Zhang et al., 2011). A customer becomes satisfied and goes for a repeat purchase if the needs and demands are fulfilled (Bisui et al., 2021). Customer loyalty is considered as repetitive behavior that results from psychological decision-making and evaluation processes (Jacoby & Kyner, 1973; Uslu & Eren, 2020), and is characterized by repeat customers (Uslu & Eren, 2020). A highly loyal customer repeatedly purchases a product/service and possesses a positive sense of attitudinal loyalty toward the brand (Pritchard & Howard, 1997). Many studies have proved that customer loyalty is considerably and positively associated with customer satisfaction (e.g., Back & Parks, 2003; Bisui et al., 2021; W. Kim et al., 2010; Uslu & Eren, 2020; Yüksel & Yüksel, 2003). Based on this it is affirmed:

*H4: Corporate brand has a positive effect on customer repurchase behavior.*
*H5: Celebrity chef has a positive effect on customer repurchase behavior.*
*H6: Customer satisfaction has a positive effect on customer repurchase behavior and has a mediation effect in the relationships among corporate brand, celebrity chef, and customer repurchase behavior.*

### Recent Studies on the Relationship Between Celebrity Chefs and Repurchase Behavior

Celebrity chefs have been a ubiquitous marketing tool utilized in the food service industry (Y. S. Chen et al., 2017). Relifra et al. (2023) indicated that celebrity endorsement has a significantly positive effect on purchasing decisions. The celebrity chefs’ endorsement on social media can also help to promote by giving attractiveness and customer engagement (García-León & Teichert, 2023). Some studies show that celebrity chefs are highly influential in raising ethical food awareness through the media (Lewis & Huber, 2015; Şahin & Gök Demir, 2023). Jin et al. (2023) illustrated that when the restaurant owner offered a celebrity chef’s service, customers were more likely to have deeper impression. Y. S. Chen et al. (2017) highlight the importance of celebrity chefs on customers’ WTP and the likelihood of repurchasing. Ilicic and Webster (2016) confirmed that celebrity brands can positively influence consumer intentions to purchase an endorsed brand. Based on that, it is proposed:

*H7: Previous consumption experience has a positive effect on the corporate brand.*

## Insight Into Our Methodological Approach

### Research Framework and Hypotheses Development

**Figure 1.**
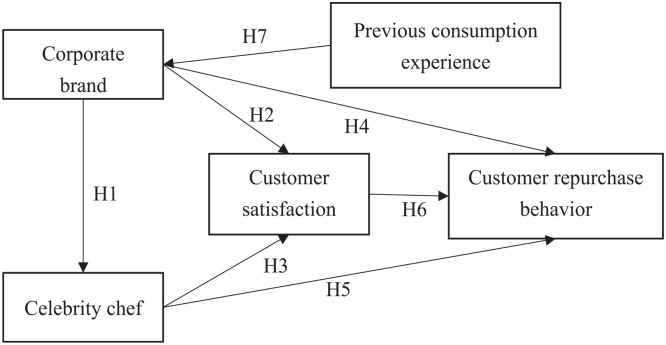
Research framework.

This study explores the incremental effect on corporate business performance through introducing personal brands of celebrity chefs in the F&B industry. A corporate brand of the F&B industry itself can affect the personal brand of the celebrity chef, and both can affect customer satisfaction and customer repurchase behavior. Customer satisfaction is used as an intermediary variable of customer repurchase behavior; on the other hand, a consumer’s previous consumption experience will impact the corporate brand. In addition, because different customers’ attributes should present different considerations when making purchase decisions, this study uses five personal attributes as control variables and these five characteristics include demographics (gender and age), human capital (education), and economic characteristics (occupation and income). Figure 1 illustrates the study’s research framework.

As mentioned above, in light of the proposed notions we put forward the following hypotheses.

*H1*: Corporate brand has a positive effect on celebrity chef.
*H2*: Corporate brand has a positive effect on customer satisfaction.
*H3*: Celebrity chef has a positive effect on customer satisfaction and has a mediation effect in the relationship between corporate brand and customer satisfaction.
*H4*: Corporate brand has a positive effect on customer repurchase behavior.
*H5*: Celebrity chef has a positive effect on customer repurchase behavior.
*H6*: Customer satisfaction has a positive effect on customer repurchase behavior and has a mediation effect in the relationships among corporate brand, celebrity chef, and customer repurchase behavior.
*H7*: Previous consumption experience has a positive effect on the corporate brand.

### Questionnaire and Data

In this study we deal with a questionnaire survey and collect quantitative data in a standardized manner to ensure that it is internally consistent and coherent for the use of analytical procedures (Durdyev et al., 2018). First, the questionnaire was designed to analyze four constructs: corporate brand, celebrity chef, customer satisfaction, and repurchase behavior (see Table 1). The main purposes of this study are to understand the impact of consumer information and services from a corporate in the purchase decision process and to understand consumers’ satisfaction with the service of a celebrity chef and the personal characteristics of the celebrity chef. This study aims to explore whether customers are willing to choose the same corporate brand again. Finally, all surveys record a range of respondent-specific characteristics in the same way. The questionnaire is designed based on the consumer’s latest purchase experience and uses a Likert 5-point scale with 64 questions. The primary data were collected from the respondents through online questionnaire in Taiwan to get 245 respondents as a sample size of the research from Nov. 10 to Nov. 25 in 2021, and through validity and reliability analysis that processed by statistical software using factor analysis and structural equation modeling. The raw data was organized in Microsoft Excel before transferring it to IBM SPSS Statistics 23.0 and AMOS version 24.0.

**Table 1.** Research Construct Definition, Items and Brief Description.

Constructs	Items	Brief description
Corporate brand	CB1	Having a good WOM
	CB2	Providing a unique service/goods
	CB3	Positive customers’ reviews
	CB4	Products are unique but expensive
	CB5	Products’ quality is good
	CB6	Buying products is convenient
	CB7	Products are better than those of other corporations
	CB8	Customer type
	CB9	Pricing strategy
	CB10	Advertisement/Promotion
	CB11	Clear brand positioning
	CB12	Higher market share
	CB13	Good sales channel
	CB15	Sound financial structure
Customer satisfaction	S5	Crisis-solving ability
	S6	Language abilities
	S7	Communication presentation skills
	S8	Planning and control abilities
	S9	Customer-oriented
	S10	Professional competence
	S11	Empathy
	S12	Prompt response
	S13	Compassion
	S14	Caring and Considerate service
	S15	Proactive attitude
	S16	Trustworthy and authenticity
Celebrity chef	P1	Persuasiveness
	P2	Attractive and charismatic
	P3	Professional skills
	P4	Trustworthy
	P5	Stable emotion
	P6	Enthusiastic and open-minded
	P8	Well preparation
	P10	Respect everyone’s difference
	P11	Strong personal-style catering philosophy
	P12	Self-confidence
	P13	Esthetics
	P14	Creativity and innovation.
	P15	Willing to share own thought
Repurchase behavior	LY1	Willing to buy continually.
	LY3	Willing to recommend families and friends
	LY4	Willing to buy regardless of price with stable quality
	LY5	Customer loyalty

## Results

### Descriptive Statistics Analysis of the Respondents

Demographic data are categorized as gender, age, education, occupation, and income level. Descriptive statistics analysis of the respondent illustrates in Table 2.

**Table 2.** Descriptive Statistics Analysis. (*N* = 245).

	Category	Frequency (*N*) and %
Gender	Female	108 (44.1%)
	Male	137 (55.9%)
Age	<24	35 (14.3%)
	24–29	75 (30.6%)
	30–35	5 (2.0%)
	36–40	5 (2.0%)
	41–46	19 (7.8%)
	>46	106 (43.3%)
Education	High School	30 (12.2%)
	University/College	125 (51.0%)
	Master’s/Doctorate	90 (36.8%)
Occupation	With jobs	163 (66.6%)
	Student	65 (26.5%)
	None	17 (6.9%)
Income per month	< NT$20,000	70 (28.9%)
	NT$20,001-30,000	52 (21.4%)
	NT$30,001-40,000	31 (12.8%)
	NT$40,001-50,000	21 (8.6%)
	NT$70,001-100,000	15 (6.1%)
	>NT$100,001	12 (4.9%)

### Reliability and Validity

#### Reliability Analysis

Cronbach’s α reliability test is a fundamental statistic to determine the reliability based on internal consistency (Churchill, 1979), and Hinton et al. (2004) recommended a minimum Cronbach’s α of .6 for a study. In general, the reliability is weak below .6, and when the value is more than .8, it is excellent (Sekaran, 2003). Therefore, we use Cronbach’s α and items to total correlation along with composite reliability to judge the reliability of the questionnaire. The composite reliability of the latent variables is composed of all observed reliability, and it presents the Cronbach’s α of all four constructs (corporate brand, celebrity chef, customer satisfaction, and repurchase behavior). Cronbach’s reliabilities for all scales are respectively .903, .904, .957, and .927, which are above the recommended threshold of .9 (Raykov, 1997). Thus, all measures indicate the scales’ internal consistency.

#### Confirmatory and Factor Analyses (CFA)

Before composing the scales for hypothesis testing, this study uses CFA to assess the construct validity of the measures and to screen the overall items to ensure that there are no duplicate or unnecessary options. After the overall analysis of the model, we delete eight items with too high MI, eventually employing 44 measurable variables for the study. At the same time, we carry out the Bartlett spherical test, and the KMO value is 0.908 (>0.9), indicating that the appropriateness of modulus sampling is good (Kaiser, 1981).

#### Analysis of Convergent Validity and Discriminant Validity

This study employs composite reliability (CR) and average variance extracted (AVE) to examine convergent validity and uses correlation analysis to test for the discriminant validity of the whole model. Discriminant validity evaluates the extent to which each construct used in the model differs from the others (Bagozzi et al., 1991). From Table 3, the CR statistics of the four constructs, respectively 0.919, 0.891, 0.953, and 0.897, exceed the recommended threshold of 0.70 (Fornell & Larcker, 1981; Hair et al., 2006). The AVE tests for sufficient discriminant validity of the constructs, and each construct has an acceptable value above 0.50 (Fornell & Larcker, 1981). Moreover, the square roots of the AVE of celebrity chef (0.715), corporate brand (0.607), customer satisfaction (0.781), and repurchase behavior (0.828) are greater than the correlations between the constructs. Support for discriminant validity of all constructs is found by comparing the square root of AVE with the correlations between constructs (Anderson & Gerbing, 1988; Fornell & Larcker, 1981). Consequently, all measures exceed the recommended threshold for convergent validity and discriminant validity.

**Table 3.** Analysis of Convergent Validity and Discriminant Validity.

Constructs	CR	AVE	Correlation coefficient			
			Celebrity chef	Corporate brand	Customer satisfaction	Repurchase behavior
Celebrity chef	0.919	0.511	1.00	-	-	-
Corporate brand	0.891	0.468	0.49	1.00	-	-
Customer satisfaction	0.953	0.609	0.63	0.40	1.00	-
Repurchase behavior	0.897	0.686	0.64	0.47	0.67	1.00

### Model Structure Analysis

#### Model Goodness-of-Fit Analysis

This study employs 13 goodness-of-fit indices and the results appear in Table 4. Although there is one index value that is not good (not below the unacceptable range), in general, the overall model is still considered good.

**Table 4.** Test for Goodness-of-Fit.

Fit measure	Test results	Acceptable	Threshold levels
*X* ^2^	1802.072		Not good
*X*^2^/*df*	1.775	1–5	Good
GFI	0.771	>0.90	Acceptable
RMR	0.067	<0.08	Good
RMSEA	0.056	<0.06	Good
AGFI	0.735	>0.9	Acceptable
NFI	0.8	>0.9	Acceptable
NNFI	0.889	>0.9	Good
CFI	0.9	>0.9	Good
RFI	0.778	>0.9	Acceptable
IFI	0.901	>0.9	Good
PNFI	0.72	>0.5	Good
PGFI	0.81	>0.5	Good

#### SEM Analysis

This study employs 44 measurable variables (X1–X40, Y1–Y4), three exogenous latent variables (F1–F3), and one endogenous latent variable (F4).

**Figure 2.**
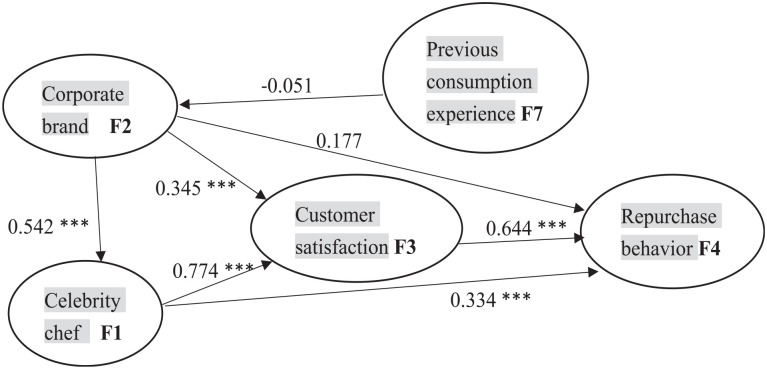
SEM analysis. *Note*. *** indicates significance at 1%.

Because the data collection of this study is based on the questionnaire, there may exist the problem of Common Method Variance (CMV). To ensure the accuracy of the research results, CMV is controlled during structural equation model analysis. The adjusted results of the analysis are in Table 5. Figure 2 illustrates SEM results.

**Table 5.** The Result of Adjusted CMV.

Path CMV	Before controlling CMV	After controlling CMV
Corporate brand → Celebrity chef	0.616***	0.542***
Corporate brand → Customer satisfaction	0.235*	0.774***
Celebrity chef → Customer satisfaction	0.798***	0.345***
Corporate brand → Repurchase behavior	0.368**	0.17
Celebrity chef → Repurchase behavior	0.484***	0.34***
Customer satisfaction → Repurchase behavior	0.528***	0.644***
RMSEA	0.062	0.056

*Note*. ***, **, and * indicate significance at 1%, 5%, and 10%, respectively.

## Discussion and Managerial Implications

### Discussion

The verification results of the seven hypotheses obtained after SEM analysis in this study are explained as follows.

(1) *Corporate brand has a positive effect on celebrity chef*.
  The results show that the influence of corporate brand on celebrity chef is significant and has a high degree of positive influence. However, the role of a celebrity chef on corporate brand is not significant, and so there is only a one-way influence between these two.
(2) *Corporate brand has a positive effect on customer satisfaction*.
  The results show that corporate brands do have a significantly positive influence on customer satisfaction. Thus, corporates should actively improve and strengthen their brand image by implementing effective and proactive marketing strategies.
(3) *Celebrity chef has a positive effect on customer satisfaction and has a mediation effect in the relationship between corporate brand and customer satisfaction*.
  The results illustrate that the positive influence of a celebrity chef on customer satisfaction is extremely significant, and the influence of corporate brand on customer satisfaction is also significant. Therefore, the direct and indirect effects of corporate brand on customer satisfaction exist, and the coefficient of celebrity chef (0.805) is much larger than that of corporate. The coefficient of corporate brand (0.235) is partly intermediary.
(4) *Corporate brand does not have a positive effect on customer repurchase behavior*.
  The result shows that corporate brand does not have a significantly positive effect on customer repurchase behavior. Hence, the effect of corporate branding on repurchase behavior is mediated by celebrity chefs, which also shows that corporate business performance may influence the primary impression of the corporate in the minds of customers.
(5) *Celebrity chef has a positive effect on customer repurchase behavior*.
  The results demonstrate that a celebrity chef can positively influence repurchase behavior, this effect is strongly significant, and a celebrity chef can mediate the effect of the corporate brand on repurchase behavior, which also shows that when a celebrity chef exists, the effect is greater than the original corporate brand. Therefore, corporates should leverage celebrity chefs’ personal brands and further assist them to develop personal brands to strengthen the corporate’s overall operation synergy.
(6) *Customer satisfaction has a positive effect on customer repurchase behavior and has a mediation effect in the relationships among between corporate brand, celebrity chef, and customer repurchase behavior*.
  The results indicate that customer satisfaction has a significantly positive effect on customer repurchase behavior, which is in line with Oliver (1999). For the part of the intermediary effect, customer satisfaction can completely mediate the effect of corporate brand on repurchase behavior. However, although a celebrity chef has a significantly positive impact on repurchase behavior, it is only partial intermediary.
(7) *Previous consumption experience does not have a positive effect on corporate brand*.
  The results reveal that the time of a customer’s latest consumption has a negative impact on the corporate brand. In other words, the longer the time is since the customer’s latest consumption, the weaker is the perception of the corporate’s brand image, and there may be even a negative effect.

### Conclusions and Managerial Implications

This research offers key inputs for different stakeholders including scholars, practitioners, and marketers. The outcomes of this study can also be used to generate various imperatives for both academics and practitioners. This study’s academic contribution is to fill up the research gap focusing on the relationship between the celebrity chef and intentions to repurchase, especially during the epidemic. In today’s marketing field, the adoption of a celebrity chef is still a popular type of strategic marketing. This study explores whether companies can improve their levels of original customer satisfaction and customer repurchase behavior after introducing the personal brand of a celebrity chef, and whether the corporate brand itself has an influence on the personal brand of a celebrity chef. We also examine the relationship between corporate brands and customers repurchase behavior. The findings show that brand does have a positive effect on customer satisfaction and customer repurchase behavior and are in line with the findings of past studies (e.g., Y. S. Chen et al., 2017; Ilicic & Webster, 2016; Jin et al., 2023; McCormick, 2016). Indeed, corporate brand does enhance the personal brand of a celebrity chef and celebrity chefs help corporate to have a positive effect on customer repurchase behavior.

The global panic associated with COVID-19 may have consequences that last for a long time on the F&B industry. For this industry, it is necessary to implement more effective marketing strategies to boost consumers’ confidence and to help businesses recover promptly in order to transform adversity into opportunity following` this global public health crisis. The results show that the existence of a celebrity chef can bring stable benefits to the corporate entity, because a good personal brand of a celebrity chef can strengthen customer loyalty. The F&B industry can enhance its resilience and sustainability by satisfying diverse consumption needs and making efforts to follow market demand trends, such as contactless services and co-brand development between a corporate and a celebrity chef to help increase sales and profit margins.

Some recent trends may indicate new possibilities for future research designs that contain both rigor and relevance. Perhaps the different social media could be used to permit more experimentation while generating more representative samples. Besides, future research can exam the difference of business performance before and after COVID-19 epidemic using event study methods etc. Furthermore, the other direction of future research is to consider the different types of celebrity chefs’ brands such as personal brand, co-brand (celebrity chef partnered with corporate) whether they have the difference on the business performance or not.
